# Pair correlations of Halton and Niederreiter Sequences are not Poissonian

**DOI:** 10.1007/s00605-021-01531-x

**Published:** 2021-02-13

**Authors:** Roswitha Hofer, Lisa Kaltenböck

**Affiliations:** Institute of Financial Mathematics and Applied Number Theory, Johannes Kepler University Linz, Altenbergerstraße 69, 4040 Linz, Austria

**Keywords:** Poissonian pair correlations, Halton sequences, Niederreiter sequences, Low-discrepancy sequences, 11K36, 11K99

## Abstract

Niederreiter and Halton sequences are two prominent classes of higher-dimensional sequences which are widely used in practice for numerical integration methods because of their excellent distribution qualities. In this paper we show that these sequences—even though they are uniformly distributed—fail to satisfy the stronger property of Poissonian pair correlations. This extends already established results for one-dimensional sequences and confirms a conjecture of Larcher and Stockinger who hypothesized that the Halton sequences are not Poissonian. The proofs rely on a general tool which identifies a specific regularity of a sequence to be sufficient for not having Poissonian pair correlations.

## Introduction

Let $$\Vert \cdot \Vert $$ denote the distance to the nearest integer. A sequence $$(x_n)_{n\ge 0}$$ of real numbers in $$[0,1)$$ has Poissonian pair correlations if

1 $$\begin{aligned} \frac{1}{N} \#\left\{ 0 \le n \ne l \le N-1 : \Vert x_n - x_l\Vert \le \frac{s}{N}\right\} \rightarrow 2s \end{aligned}$$

for every real number $s \ge 0$ as $N \rightarrow \infty$.

The investigation of pair correlations of sequences was originally motivated by problems in quantum chaos, see e.g. [1] and the references therein. In the last few years pair correlations have also been studied from a purely mathematical point of view as the property of Poissonian pair correlations is natural for a sequence of independently chosen random numbers drawn from the uniform distribution. Extensive research recently has been done in terms of metrical theory as well as for concrete sequences. An introduction to this topic and a collection of results is provided by [13].

For example, it is known that any sequence $$(x_n)_{n\ge 0}$$ in $$[0,1)$$ which has Poissonian pair correlations is also uniformly distributed, i.e.

$$\begin{aligned} \lim _{N \rightarrow \infty } \frac{1}{N} \#\{0 \le n \le N -1: x_n \in [a,b)\} = b-a \end{aligned}$$

for all $0 \le a < b \le 1$ (this was independently proven in [2, 7, 21]). However, the converse is not true since for many explicit examples of classical uniformly distributed sequences, such as the Kronecker sequence $(\{n\alpha \})_{n\ge 0}$, the van der Corput sequence and certain digital (*t*, 1)-sequences, it has been shown that they do not have Poissonian pair correlations (see e.g. [14]).

A generalization of Poissonian pair correlations to a higher-dimensional setting has recently been established in [9] as well as in [22] (see also [16] for a more general analysis of higher-dimensional pair correlations). In this work, we refer to the concept in [9]. To this end, let $\Vert \cdot \Vert _\infty$ denote a combination of the supremum-norm of a *d*-dimensional vector $\varvec{x} = (x^{(1)}, \dots , x^{(d)}) \in \mathbb {R}^d$ with the distance to the nearest integer function $\Vert \cdot \Vert$ defined by

2 $$\begin{aligned} \Vert \varvec{x} \Vert _\infty := \max (\Vert x^{(1)}\Vert , \dots , \Vert x^{(d)}\Vert ). \end{aligned}$$

A *d*-dimensional sequence $(\varvec{x}_n)_{n \ge 0}\in [0,1)^d$ has Poissonian pair correlations if

3 $$\begin{aligned} \frac{1}{N} \#\left\{ 0 \le n \ne l \le N-1 : \Vert \varvec{x}_n - \varvec{x}_l\Vert _{\infty } \le \frac{s}{N^{1/d}}\right\} \rightarrow (2s)^d \end{aligned}$$

for every real number $s \ge 0$ as $N \rightarrow \infty$.

The alternative higher-dimensional concept that was introduced in [22] is based on the Euclidean norm instead of the maximum in (2). The specific form on the left hand side in (3) can be explained by regarding the one-dimensional case (1) first. For a regular grid with *N* points on the one-dimensional torus together with the setting $s=1$, we know that every point counts exactly the two neighboring points in its closed 1/*N*-neighborhood and therefore the left hand side of (1) equals 2, which equals the right hand side of (1) for $s=1$. Now a one-dimensional sequence with Poissonian pair correlations has to satisfy for each *s* that the expected number of points in a closed *s*/*N*-neighborhood equals 2*s* in the limit. In the higher-dimensional definition the neighborhood is the closed ball with diameter $2s/N^{1/d}$ and the expected number of points in such a closed neighborhood has to be equal to the volume of the ball with diameter 2*s*.

Any sequence which fulfills (3) gives the impression of being very evenly distributed on the torus. In fact, in analogy to the one-dimensional case, it could be shown that sequences with the Poissonian pair correlation property in the sense of [9] as well as [22] are uniformly distributed in $$[0,1)^d$$. Again in analogy to the one-dimensional case, the converse is not true for higher-dimensional sequences. For example, the *d*-dimensional Kronecker sequence $$(\{n\varvec{\alpha }\})_{n\ge 0}$$ does not have Poissonian pair correlations in the sense of (3) for any $$\varvec{\alpha } \in \mathbb {R}^d$$ (see [9]) in consistency with the fact that the one-dimensional Kronecker sequence $$(\{n{\alpha }\})_{n\ge 0}$$ does not have Poissonian pair correlations for any $$\alpha \in \mathbb {R}$$. However, whether the higher-dimensional analogues of other well-distributed one-dimensional point sequences—such as Halton or digital (*t*, *d*)-sequences—have Poissonian pair correlations remained unanswered although it is not so hard to disprove Poissonian pair correlations for their one-dimensional versions (see e.g. [14]).

One might conjecture that for Poissonian pair correlations in the higher-dimensional setting it may be a necessary condition that all its component sequences do have Poissonian pair correlations. In other words, is the fact that a single component is not Poissonian sufficient to disprove Poissonian pair correlations for the higher-dimensional sequence? We are not aware of an argument that leads to a transfer of the non-Poissonian pair correlation property from one or more components to the higher-dimensional sequence. The higher-dimensional version of uniform distribution that conditions for a sequence $$(\varvec{x}_n)_{n \ge 0}$$ in $$[0,1)^d$$,

$$\begin{aligned} \lim _{N\rightarrow \infty } \frac{1}{N}\# \left\{ 0\le n\le N-1: \varvec{x}_n\in \prod _{j=1}^d[a_j,b_j)\right\} =\prod _{j=1}^d(b_j-a_j) \end{aligned}$$

for all $0\le a_j<b_j\le 1$ and $j\in \{1,\ldots ,d\}$ includes uniform distribution for every component sequence.

The motivation behind the present paper was to prove that both the Halton sequences and the Niederreiter sequences do not have Poissonian pair correlations in the sense of (3). We emphasize that it is not ad hoc clear whether the two higher-dimensional concepts of Poissonian pair correlations in [9] and [22] are equivalent or not. Therefore the first task for the investigation is to choose one of the concepts in higher dimensions. We use (3), because it has been used for the investigation of the higher-dimensional Kronecker sequence and it appears more suitable for the investigations of the Halton as well as digital (*t*, *d*)-sequences, because in the proof we can reduce the study of the distances between points to one single component.

Before the presentation of our results, let us briefly review the construction of the considered sequences.

### Digital (*t*, *d*)-sequences

A widely used class of low-discrepancy sequences for numerical integration methods are digital (*t*, *d*)-sequences that are generated via the digital method:

* Let* $d \in \mathbb {N}$, *let* $\mathbb {F}_q$ *be a finite field with* *q* *elements and characteristic* *p* *and let* $\phi : \mathbb {F}_q \rightarrow \{0, 1, \dots , q-1\}$ *be a bijection satisfying* $\phi (0)=0$. *The main ingredients are the* $\mathbb {N}\times \mathbb {N}_0$ *generating matrices* $C^{(1)},\ldots , C^{(d)}$ *over the finite field* $\mathbb {F}_q$ *with row index range* $\mathbb {N}$ *and column index range* $\mathbb {N}_0$. *We construct a sequence* $(\varvec{x}_n)_{n \ge 0}$, $\varvec{x}_n = (x_n^{(1)}, \dots , x_n^{(d)})$, *by generating the* *j-th component of the* *n-th point,* $x_n^{(j)}$, *as follows. We represent* $n = n_0 + n_1q + n_2q^2 + \cdots$ *in base* *q*, *build the vector* $(\phi ^{-1}(n_0), \phi ^{-1}(n_1), \dots )^\top \in \mathbb {F}_q^{\mathbb {N}_0}$ *with infinitely many entries, set*

$$\begin{aligned} C^{(j)} \cdot (\phi ^{-1}(n_0), \phi ^{-1}(n_1), \dots )^\top =: (y^{(j)}_1, y^{(j)}_2, \dots )^\top \in \mathbb {F}_q^{\mathbb {N}} \end{aligned}$$

*and*

$$\begin{aligned} x_n^{(j)} := \sum _{i \ge 1} \frac{\phi (y_i^{(j)})}{q^{i}}. \end{aligned}$$

*Note that the matrix vector multiplication above is well defined, since the vector* $(\phi ^{-1}(n_0), \phi ^{-1}(n_1), \dots )^\top$ *contains only finitely many nonzero entries. *

The distribution properties of the constructed sequence $(\varvec{x}_n)_{n \ge 0}$ strongly depend on the generating matrices $C^{(1)},\ldots , C^{(d)}$. For the sake of simplicity we assume that all matrices have finite columns, i.e., each column contains only a finite number of non-zero entries.

If there exists a number $t \in \mathbb {N}_0$ such that for every $m \in \mathbb {N}$ with $m>t$ and for all $r_1, \dots , r_d \in \mathbb {N}_0$ with $r_1 + \dots + r_d = m-t$, the set of row vectors

$$\begin{aligned} \left\{ (c^{(j)}_{r,k})_{0 \le k < m}: j \in \{1, \dots , d\}, r \in \{1, \dots , r_j\}\right\} \end{aligned}$$

is linearly independent over $\mathbb {F}_q$, then $(\varvec{x}_n)_{n \ge 0}$ is a (*t*, *d*)-sequence in base *q*, i.e. for all integers $m > t$ and $s \in \mathbb {N}_0$ the point set $(\varvec{x}_n)_{sq^m \le n < (s+1)q^m}$ has the property that any elementary interval of order $m-t$, that is an interval of the form

$$\begin{aligned} I(v_1, \dots , v_d) := \prod _{j = 1}^d \Big [\frac{a_j}{q^{v_j}},\frac{a_j+1}{q^{v_j}}\Big ) \end{aligned}$$

with $v_1,\ldots ,v_d\in \mathbb {N}_0$ such that $v_1 + \dots + v_d = m-t$ and with $a_j \in \{0,1, \dots , q^{v_j}-1\}$ for every$j\in \{1,\ldots ,d\}$, contains exactly $q^t$ points. For more detailed information on (*t*, *d*)-sequences and their construction we refer to [3, 19] and the references therein.

In order to reflect a deeper regularity in the single components the more specific notion of $(u, \varvec{e}, d)$-sequences, where $u \in \mathbb {N}_0$ and $\varvec{e}= (e_1, \dots , e_d)\in \mathbb {N}_0^d$, was introduced by Tezuka in [24] and analyzed e.g. in [11]. For constructing a $(u, \varvec{e}, d)$-sequence the generating matrices $C^{(1)},\ldots , C^{(d)}\in \mathbb {F}_q^{\mathbb {N}\times \mathbb {N}_0}$ have to satisfy for all $r_1, \dots , r_d \in \mathbb {N}_0$ such that $e_1r_1 + \dots + e_dr_d \le m-u$, the set of row vectors

$$\begin{aligned} \left\{ (c^{(j)}_{r,k})_{0 \le k < u + \sum _{j=1}^{d}e_jr_j}: j \in \{1, \dots , d\}, r \in \{1, \dots , e_jr_j\}\right\} \end{aligned}$$

has to be linearly independent over $\mathbb {F}_q$. Then, for all integers $m > u$ and $s \in \mathbb {N}_0$ the point set $(\varvec{x}_n)_{sq^m \le n < (s+1)q^m}$ satisfies that for any $v_1, \dots , v_d \in \mathbb {N}_0$ with $e_1v_1 + \dots + e_d v_d \le m-u$, the elementary interval $I(e_1v_1, \dots , e_dv_d)$ contains exactly $q^{m - (e_1v_1 + \dots + e_d v_d)}$ points.

It is known that any $(u,\varvec{e},d)$-sequence in base *q* is a (*t*, *d*)-sequence in base *q* with $t = u + \sum _{j = 1}^d (e_j-1)$. Tezuka [24] showed that for the Niederreiter sequences [18] an appropriate choice of $\varvec{e}$ allows smallest possible quality parameter $u=0$ and stresses a deeper regularity of the Niederreiter sequences that will come in handy for the study of their pair correlations.

It is a non-trivial task to find or construct matrices satisfying such strict conditions on their rank structure. The known methods are either constructions that determine the generating matrices row by row (as e.g. the generating matrices of Niederreiter sequences [18] and of Xing-Niederreiter sequences [26]) or column-by-column construction algorithms (as e.g. the generating matrices introduced in [10, 11]).

For the question whether any digital (*t*, *d*)-sequence can have Poissonian pair correlations or not the investigation of both concepts is relevant. In this paper we study the most prominent example of the row-by-row concept by Niederreiter [18] and the most basic form of the column-by-column concept [10]. This two classes of digital sequences are generated by non-singular upper triangular (*NUT*) matrices and satisfy $u=0$ for appropriate $\varvec{e}$. Both properties, *NUT* and $u=0$, are used in our method of proof.

The generating matrices of Niederreiter [18]: * for a given dimension* $d \in \mathbb {N}$ *we choose* $q_1(x), \dots , q_d(x) \in \mathbb {F}_q[x]$ *to be monic non-constant pairwise co-prime polynomials over* $\mathbb {F}_q$ *of degrees* $e_j:=\deg {q_j(x)} \ge 1$ *for* $j \in \{1, \dots , d\}$. *Set* $\varvec{e}=(e_1, \dots , e_d)$. *Now the* *i-th row of the* *j-th generating matrix* $C^{(j)}$, *denoted by* $\rho ^{(j)}_i$, *is constructed as follows. We choose* $s \in \mathbb {N}$ *and* $r \in \{0, \dots , e_j-1\}$ *such that* $i = e_j s - r$, *consider the expansion*

$$\begin{aligned} \frac{x^r}{q_j(x)^s}=\sum _{k \ge 0} a^{(j)}(s,r,k)x^{-k-1} \in \mathbb {F}_q((x^{-1})) \end{aligned}$$

*and set* $$\rho ^{(j)}_i=(a^{(j)}(s,r,k))_{k\ge 0}$$.

It is easy to check that these generating matrices are *NUT* matrices over $\mathbb {F}_q$. Furthermore, they generate a digital $(0,\varvec{e},d)$-sequence over $\mathbb {F}_q$ (see e.g. [24]).

The analog generating matrices that are constructed column-by-column [10]: *for a given dimension* $d\in \mathbb {N}$ *we choose* *d* *pairwise co-prime, monic non-constant polynomials* $q_1(x), \dots , q_d(x) \in \mathbb {F}_q[x]$ *and denote their degrees, which are all positive, by* $e_1,\ldots ,e_d$. *For* $k \in \mathbb {N}_0$, *we construct the* *k-th column of the* *j-th generating matrix* $C^{(j)}$, *denoted by* $\sigma ^{(j)}_k = (\sigma ^{(j)}_{t,k})_{t \ge 1}$, *by using the representation of* $x^{k}$ *in terms of powers of* $q_j(x)$, *i.e.,* $x^k = \sum _{s\ge 0} b_s(x) q_j^s(x)$ *with* $b_s(x) \in \mathbb {F}_q[x]$ *satisfying* $\deg (b_s(x)) < e_j$. *This representation can be computed as follows:*

$$\begin{aligned} x^k&= a_0(x) q_j(x)+b_0(x),\quad \text { where } a_0(x), b_0(x) \in \mathbb {F}_q[x] \text { such that } \deg {b_0(x)}< e_j,\\ a_0(x)&= a_1(x) q_j(x)+b_1(x),\quad \text { where } a_1(x), b_1(x)\in \mathbb {F}_q[x] \text { such that } \deg {b_1(x)} < e_j,\\&\,\,\, \vdots \,\,\, . \end{aligned}$$

*Note that there are just finitely many nonzero remainder polynomials* $b_s(x)$. *Now we consider the representation of the remainder polynomial* $b_s(x)$ *in terms of powers of* *x*, *i.e.* $b_s(x) = b_{s,0} + b_{s,1}x + \dots + b_{s,e_j - 1}x^{e_j - 1}$, *and set*

$$\begin{aligned} (\sigma ^{(j)}_{e_js+1,k}, \sigma ^{(j)}_{e_js+2,k}, \dots , \sigma ^{(j)}_{e_js+e_j,k}):= (b_{s,0},b_{s,1}, \dots , b_{s,e_j-1}). \end{aligned}$$

The matrices $C^{(1)}, \dots , C^{(d)}$ are *NUT* matrices and generate a $(0, \varvec{e}, d)$-sequence in base *q* (cf. [10, proof of Theorem 1]).

For both methods—the Niederreiter construction as well as the column-by-column construction—we strongly focus on analyzing the left hand side of (3) in order to obtain our first main result.

#### Theorem 1

The digital $(0, \varvec{e}, d)$-sequences with generating matrices $C^{(1)}, \dots , C^{(d)}$ obtained via the Niederreiter construction or the alternative column-by-column approach do not have Poissonian pair correlations.

Note that both methods of constructing generating matrices based on the specific choice of distinct monic linear polynomials $q_1(x),\ldots ,q_d(x)$ result in the generating matrices of the Faure sequences [4]. Hence, we know that Faure sequences do not have Poissonian pair correlations. Moreover, we would like to note that the proof of Theorem 1 utilizes the *NUT* property of the generating matrices as well as polynomial arithmetic over $\mathbb {F}_q$. It is a non trivial task to generalize the method of proof of Theorem 1 to more general not necessarily upper triangular generating matrices, i.e. to cover the generalized Niederreiter sequences as well as the Sobol sequences, and also the Hofer–Niederreiter sequences [11] and Xing–Niederreiter sequences [26].

### Halton sequences

Other higher-dimensional point sequences which are of wide interest and which can be seen as the extension of the van der Corput sequence to higher dimensions are Halton sequences [8].

* Let* $d \in \mathbb {N}$, $b_1, \dots , b_d\ge 2$ *be pairwise relatively prime integers and for* $b\ge 2$ *let* $\phi _b: \mathbb {N}_0 \rightarrow [0,1)$ *be the* *b-adic radical inverse function, defined as*

$$\begin{aligned} \phi _b(n) := \frac{n_0}{b} + \frac{n_1}{b^2} + \cdots \end{aligned}$$

*where* $$n = n_0 + n_1b + \dots $$ *with* $$n_i \in \{0, \dots , b-1\}$$ *for* $$i \in \mathbb {N}_0$$ *is the unique base* *b* *representation of* *n*. *The Halton sequence in pairwise relatively prime integer bases* $$b_1, \dots , b_d\ge 2$$ *is the sequence* $$(\varvec{x}_n)_{n \ge 0}$$ *in* $$[0,1)^d$$ *whose elements are given by*

$$\begin{aligned} \varvec{x}_n = (\phi _{b_1}(n), \dots , \phi _{b_d}(n)). \end{aligned}$$

Again, see e.g. [3] for more details. The question whether Halton sequences have Poissonian pair correlations or not was posed in [9] and also stated as Problem 5 in [13], although it was suggested that this is most likely not the case. It turns out that this conjecture indeed is true.

#### Theorem 2

The Halton sequence $(\varvec{x}_n)_{n \ge 0}$ in pairwise relatively prime integer bases $b_1, \dots , b_d\ge 2$, $d \in \mathbb {N}$, does not have Poissonian pair correlations.

Of course, it typically is expected that higher-dimensional versions of sequences have similar qualities as their one-dimensional analogues. However, it should be mentioned that an exceptional behavior of Halton sequences has been announced for the instance of the $L_p$-discrepancy for $p<\infty$ by Levin in [15]. In this unpublished manuscript he claimed that higher-dimensional Halton sequences have optimal order of $L_p$-discrepancy, even though the one-dimensional van der Corput sequence does not satisfy optimal $L_p$-discrepancy bounds. (For the result on the $L_p$-discrepancy of the one-dimensional van der Corput sequence cf. e.g. [20]. The interested reader is referred to [5], which gives an overview over different generalizations of van der Corput sequences and some of their properties.)

The rest of the paper is organized as follows. The key ingredient for the proofs of Theorem 1 and Theorem 2 is a general tool stated as Proposition 1 at the beginning of the next section. It identifies a specific high regularity for sequences which is sufficient for failing Poissonian pair correlations. In more detail, if there are too many points sharing similar distances from each other then the pair correlations cannot be Poissonian. Verifying such a high regularity for both, Niederreiter and Halton sequences, is not trivial and therefore takes the majority of Sect. 2. The final Sect. 3 gives an outlook to future research tasks and discusses a problem in algebraic number theory and Diophantine approximation that occurred during the investigation of Halton sequences.

## Proofs

The following proposition, which serves as one of our key tools for the proofs of Theorems 1 and 2, works out that any sequence that contains a sufficiently large number of pairs of points with similar distances fails to have Poissonian pair correlations.

### Proposition 1

Let $$(\varvec{x}_n)_{n \ge 0}$$ be a sequence in $$[0,1)^d$$. If there exist real numbers $$a,b,c > 0$$ satisfying

4 $$\begin{aligned} c> (2b)^d - (2a)^d>0, \end{aligned}$$

and a strictly increasing sequence of positive integers $(N_k)_{k \in \mathbb {N}}$ such that $(\varvec{x}_{n})_{0 \le n < N_k}$ fulfills

5 $$\begin{aligned} \#\left\{ 0 \le n \ne l \le N_k-1 : \Vert \varvec{x}_n - \varvec{x}_l\Vert _{\infty } \in \left( \frac{a}{N_k^{1/d}},\frac{b}{N_k^{1/d}}\right] \right\} \ge c N_k \end{aligned}$$

for all *k* larger than some index $k_0$, then $(\varvec{x}_n)_{n \ge 0}$ does not have Poissonian pair correlation.

### Proof

Let $$a,\,b,\,c>0$$ be real numbers satisfying (4). Let $$(\varvec{x}_n)_{n \ge 0}$$ be a sequences in $$[0,1)^d$$ and $$(N_k)_{k\in \mathbb {N}}$$ a strictly increasing sequence in $$\mathbb {N}_0$$ such that (5) holds for every *k* large enough.

Assume that $(\varvec{x}_n)_{n \ge 0}$ has Poissonian pair correlations. We use (3) for $s = b$ and obtain

$$\begin{aligned} \frac{1}{N_k} \#\Big \{0 \le n \ne l \le N_k-1 : \Vert \varvec{x}_n - \varvec{x}_l\Vert _{\infty } \le \frac{b}{N_k^{1/d}}\Big \} \rightarrow (2b)^d \end{aligned}$$

as $k \rightarrow \infty$. It holds that

$$\begin{aligned} \#\Big \{0&\le n \ne l \le N_k-1 : \Vert \varvec{x}_n - \varvec{x}_l\Vert _{\infty } \le \frac{b}{N_k^{1/d}}\Big \} \\&= \#\Big \{0 \le n \ne l \le N_k-1 : \Vert \varvec{x}_n - \varvec{x}_l\Vert _{\infty } \le \frac{a}{N_k^{1/d}}\Big \} \\&\quad + \#\Big \{0 \le n \ne l \le N_k-1 : \Vert \varvec{x}_n - \varvec{x}_l\Vert _{\infty } \in \Big (\frac{a}{N_k^{1/d}},\frac{b}{N_k^{1/d}}\Big ]\Big \} \\&=: A_k + B_k. \end{aligned}$$

Therefore, for any $\varepsilon _1 > 0$ there exists an index $k(\varepsilon _1)$ such that for all $k > k(\varepsilon _1)$ we have

$$\begin{aligned} \frac{A_k}{N_k} + \frac{B_k}{N_k} \le (2b)^d + \varepsilon _1. \end{aligned}$$

For sufficiently large *k* we can use the assumption (5) and obtain

$$\begin{aligned} \frac{A_k}{N_k} \le (2b)^d + \varepsilon _1 - \frac{B_k}{N_k} \le (2b)^d + \varepsilon _1 - c. \end{aligned}$$

Now consider $A_k/N_k$ which tends to $(2a)^d$ as $k \rightarrow \infty$ by the property of Poissonian pair correlations for $s = a$. Again this implies that for any $\varepsilon _2 > 0$ there is an index $k(\varepsilon _2)$ such that for all $k > k(\varepsilon _2)$ it holds that

$$\begin{aligned} \frac{A_k}{N_k} \ge (2a)^d - \varepsilon _2. \end{aligned}$$

By assumption (4), there exists $\kappa > 0$ such that

$$\begin{aligned} c = (2b)^d - (2a)^d + \kappa . \end{aligned}$$

However, if $\varepsilon _1$ and $\varepsilon _2$ are chosen such that $\varepsilon _1 + \varepsilon _2 < \kappa$ and provided that *k* is sufficiently large we have

$$\begin{aligned} (2a)^d - \varepsilon _2\le \frac{A_k}{N_k} \le (2b)^d + \varepsilon _1 - c \end{aligned}$$

and

$$\begin{aligned} c\le (2b)^d-(2a)^d+ \varepsilon _1+\varepsilon _2<(2b)^d - (2a)^d + \kappa =c, \end{aligned}$$

which yields the desired contradiction to our assumption that $(\varvec{x}_n)_{n \ge 0}$ has Poissonian pair correlations. $\square$

In the light of Proposition 1, the key ingredient for proving that Niederreiter and Halton sequences do not have Poissonian pair correlations therefore is to find enough pairs of points for which the distances between those points can be suitably well calculated and lie in a rather small interval. Note that the larger $|b-a|$, the larger *c* in (4) and the more pairs of points $(\varvec{x}_n,\varvec{x}_l)$ satisfying the counting condition in (5) have to be identified.

### Application to digital sequences

For the proof of Theorem 1 we need some preliminary results.

#### Lemma 1

[6, Prop.1] Let $C^{(1)},\ldots ,C^{(d)}$ be the generating matrices of a digital $(u,\varvec{e},d)$-sequence over $\mathbb {F}_q$ and *S* be a *NUT* matrix in $\mathbb {F}_q^{\mathbb {N}_0\times \mathbb {N}_0}$. Then $C^{(1)}S,\ldots ,C^{(d)}S$ are generating matrices of a digital $(u,\varvec{e},d)$-sequence over $\mathbb {F}_q$.

In the following the quantity $L_f$ denotes the maximal row length considering the first *f* rows of all generating matrices. More precisely, taking the matrix consisting of the first *f* rows of each of the generating matrices, $L_f-1$ is the index of the last non-zero column (or $\infty$, if none exists).

#### Lemma 2

Let $C^{(1)},\ldots ,C^{(d)}$ be the generating matrices associated to the distinct monic non-constant pairwise co-prime polynomials $q_1(x),\ldots ,q_d(x)$ with degrees $e_1,\ldots ,e_d$ using one of the two constructions given in Sect. 1.1. We set $v:=\mathrm{lcm}(e_1,\ldots ,e_d)$ and define the matrix $S\in \mathbb {F}_q^{\mathbb {N}_0\times \mathbb {N}_0}$ as follows. For $k\in \mathbb {N}_0$, the *k*-th column $\sigma _k$ of *S*, is given by $\sigma _k = (b_0, b_1, \dots , b_{k-1}, b_k, \dots )^\top$ where the $b_n$, $0\le n\le k$, are the coefficients of the following monic polynomial of degree *k*,

$$\begin{aligned} p_k(x)=x^{r_1}\prod _{i=1}^d q_i(x)^{(s_i+s_{i+1}+ \cdots +s_d)v/e_i} = \sum _{n\ge 0}b_nx^{n}. \end{aligned}$$

Here the $s_i$ and $r_i$ are defined as follows

$$\begin{aligned} k&= dv s_d+r_d,&\, r_d \in \{0,\ldots ,vd-1 \} \\ r_d&= (d-1)vs_{d-1}+r_{d-1},&\, r_{d-1}\in \{0,\ldots ,v(d-1)-1\} \\&\,\,\,\vdots&\vdots \,\,\, \\ r_2&= v s_1+r_1,&\, r_1\in \{0,\ldots ,v-1\}. \end{aligned}$$

Then the matrices $C^{(1)}S, \dots , C^{(d)}S$ generate a digital $(0,\varvec{e},d)$-sequence and satisfy $L_f\le d f$ provided that *v*|*f*.

#### Proof

For the Niederreiter construction see [12, Theorem 1]. For the column by column construction note the linearity of the construction algorithm and the fact that the set of polynomials $(1,p_1(x),p_2(x),\ldots ,p_k(x))$ forms a basis of $\{p\in \mathbb {F}_q[x]: \deg (p)\le k\}$. Moreover, note that $q_i^{(s_i+s_{i+1}+\cdots +s_d)v/e_i}(x)$ divides $p_k(x)$ and that for $k\ge d f$ we have $v s_d\ge f$. Thus for such *k* at least *df* zero entries in the *k*-th columns of $C^{(1)},\ldots ,C^{(d)}$ are guaranteed. $\square$

The matrix *S*, which was introduced in Lemma 2, will prove to be extremely useful for the sharp estimating of the distances between specific pairs of points in the following proof of Theorem 1.

#### Proof of Theorem 1

We may assume $d\ge 2$ as the one-dimensional case was treated already in [14]. Let $q_j(x)$, $j \in \{1, \dots , d\}$ be monic non-constant pairwise co-prime polynomials over $\mathbb {F}_q$ of degrees $e_j \ge 1$ and let $C^{(j)}$ denote the corresponding generating matrices constructed via the Niederreiter or the alternative column by column approach. Moreover, let *m* be a multiple of $v = \mathrm{lcm}(e_1, \dots , e_d)$ times *d*, i.e. $m = kvd$ with $k \in \mathbb {N}$,.

The sequence $(\varvec{x}_n)_{n\ge 0}$ constructed via the digital method is a $(0,\varvec{e}, d)$-sequence, therefore any elementary interval with volume $q^{-m}$ of the form

$$\begin{aligned} I(vk, \dots , vk) = \prod _{j=1}^d\left[ \frac{a_j}{q^{vk}},\frac{a_j+1}{q^{vk}}\right) \end{aligned}$$

where $0 \le a_j < q^{vk}$, contains exactly one point $\varvec{x}_n$ with $n \in \{0, 1, \dots , q^m-1\}$ and one point $\varvec{x}_l$ with $l \in \{q^m, q^m +1, \dots , 2 q^m -1\}$. The idea of the proof then is to find infinitely many suitable values of *m* such that the distances between the elements $\varvec{x}_n$ and $\varvec{x}_l$ are similar for many *n* and *l*, respectively, in order to apply Proposition 1.

To begin with, for arbitrary $n = n_0 + n_1q + \dots$ and $l = l_0 + l_1q + \dots$ we define

$$\begin{aligned} \varDelta _{n,l} := (\phi ^{-1}(l_0), \phi ^{-1}(l_1), \dots , \phi ^{-1}(l_{m}))^\top - (\phi ^{-1}(n_0), \phi ^{-1}(n_1), \dots , \phi ^{-1}(n_{m}))^\top \in \mathbb {F}_q^{m+1}. \end{aligned}$$

Note that $n_m=0$ and $l_m=1$.

The elements $\varvec{x}_n$ and $\varvec{x}_l$ lie in the same elementary interval $I(vk, \dots , vk)$ if

6 $$\begin{aligned} D_{m\times (m+1)}\varDelta _{n,l} = (0, \dots , 0)^\top , \end{aligned}$$

where $D_{m\times (m+1)} \in \mathbb {F}_q^{m \times (m+1)}$ is the $(m \times (m+1))$-matrix whose rows consist of the rows of each upper left $(kv \times (m+1))$-submatrix of $C^{(j)}$, $j \in \{1, \dots , d\}$. Note using the fact that both the Niederreiter construction as well as the column by column approach yield digital $(0,\varvec{e},d)$-sequences, we easily derive from the definition of a digital $(u,\varvec{e},d)$-sequences that the matrix $D_{m\times (m+1)}$ has rank *m*.

Furthermore, let $S_{m+1}$ be the upper left $((m+1) \times (m+1))$-submatrix of *S* defined in Lemma 2. Since $S_{m+1}$ is non-singular we can rewrite

$$\begin{aligned} D_{m\times (m+1)} \varDelta _{n,l} = D_{m\times (m+1)} S_{m+1} S^{-1}_{m+1}\varDelta _{n,l}. \end{aligned}$$

Since $0 \le n < q^m$, $q^m \le l < 2 q^m$ and both, $S_{m+1}$ and $S^{-1}_{m+1}$ are *NUT* matrices with ones in the diagonal, we have

$$\begin{aligned} S^{-1}_{m+1}\varDelta _{n,l} = \begin{pmatrix}\varvec{d}\\ \phi ^{-1}(1)\end{pmatrix} \end{aligned}$$

with $\varvec{d} \in \mathbb {F}_q^m$ and where $\phi$ is the bijection from the construction of digital (*t*, *d*)-sequences (cf. Sect. 1.1). Moreover, from Lemma 2 with $f = kv$ it follows that the last column of the product $D_{m\times (m+1)} S_{m+1}$ consists of zeros exclusively. From (6) it therefore follows that $\varvec{d}$ is the zero vector in $\mathbb {F}_q^m$, i.e.

$$\begin{aligned} S^{-1}_{m+1}\varDelta _{n,l} = \begin{pmatrix} \varvec{0} \\ \phi ^{-1}(1) \end{pmatrix}, \end{aligned}$$

or equivalently after multiplying with $S_{m+1}$ from the left, and hence the $m+1$-st component of $\varDelta _{n,l}$ equals $\phi ^{-1}(1)$ times the $(m+1)$-st column of $S_{m+1}$, which is determined by the representation of the polynomial

$$\begin{aligned} p_m(x)=\prod _{i=1}^dq^{vk/e_i}_i(x) \end{aligned}$$

in terms of powers of *x*.

We now have to find specific choices of *m* or *k*, respectively, such that we obtain special $\varDelta _{n,l}$ in order to apply Proposition 1. Therefore, let

$$\begin{aligned} \tau _i:=\min \left\{ r \in \mathbb {N}: q_i^{rv/e_i}(x) \equiv 1 \pmod {q_j^{v/e_j}(x)} \text { for every } j \ne i \right\} . \end{aligned}$$

Then we use the characteristic *p* of the finite field $\mathbb {F}_q$ and the fact that for all $k \in \mathbb {N}$ and $1 \le l < p^k$ we have $$\left( {\begin{array}{c}p^k\\ l\end{array}}\right) \equiv 0 \pmod {p}$$. So whenever *u* is a power of the characteristic of $\mathbb {F}_q$ we have for all $f(x), g(x) \in \mathbb {F}_q[x]$ that

$$\begin{aligned} (f(x)+g(x))^u=f^u(x)+g^u(x). \end{aligned}$$

If we set $\theta :=\mathrm{lcm}(\tau _1 ,\ldots ,\tau _d)$, we therefore have

$$\begin{aligned} q_i^{\theta v/e_i}(x)\equiv 1 \pmod {q_j^{v/e_j}(x)} \end{aligned}$$

and

7 $$\begin{aligned} \prod _{\begin{array}{c} i = 1 \\ i \ne j \end{array}}^{d} q_i^{uv\theta /e_i}(x)\equiv 1 \pmod {q_j^{uv/e_j}(x)}. \end{aligned}$$

Then let $m = uv\theta d$ and consider $C^{(j)}_{(m+1) \times (m+1)}\varDelta _{n,l}$.

1. If the matrix $C^{(j)}$ is constructed via the Niederreiter approach, then the *k*-th entry of $C^{(j)}_{(m+1) \times (m+1)}\varDelta _{n,l}$ is the coefficient of $x^{-1}$ in the Laurent series expansion of
  $$\begin{aligned} \phi ^{-1}(1) \frac{x^r}{q_j^s(x)} \prod _{i = 1}^{d}q_i^{uv\theta /e_i}(x), \end{aligned}$$
  with $k = e_js - r$ and $r \in \{0,1, \dots , e_j-1\}$. For $s \le uv\theta /e_j$ and any admissible value of *r*, the expression above is a polynomial and therefore the coefficient of $x^{-1}$ in its Laurent series expansion is 0. For $uv\theta /e_j < s \le uv\theta /e_j + uv/e_j$ we use (7) to get
  $$\begin{aligned} \frac{x^r}{q_j^{s}(x)}\prod _{\begin{array}{c} i = 1 \end{array}}^{d}q_i^{uv\theta /e_i}(x) = x^rb(x) + \frac{x^r}{q_j^{s-uv\theta /e_j}(x)}, \end{aligned}$$
  for some polynomial $b(x) \in \mathbb {F}_q[x]$. Remember that $x^rq_j^{-(s-uv\theta /e_j)}(x)$ exactly determines row $e_j(s-uv\theta /e_j)-r$ of $C^{(j)}$ and that the coefficient of $x^{-1}$ of this expression is the entry in the first column. Since $C^{(j)}$ is a *NUT* matrix with 1s in the diagonal we obtain
  $$\begin{aligned} C^{(j)}_{m+1 \times m+1}\varDelta _{n,l} = \left( \underbrace{0, \dots , 0}_{uv\theta }, \phi ^{-1}(1),\underbrace{0, \dots , 0}_{uv-1}, a, \dots \right) ^\top , \end{aligned}$$
  with $$a \in \mathbb {F}_q$$.
2. In case that the matrix $C^{(j)}$ is constructed via the column by column approach, the entries of $C^{(j)}_{(m+1) \times (m+1)}\varDelta _{n,l}$ are given by the coefficients of the representation of $\phi ^{-1}(1)p_m(x)$ in terms of powers of $q_j(x)$. Using (7) we get
  $$\begin{aligned} p_m(x)&= \prod _{i = 1}^{d} q_i^{uv\theta /e_i}(x) \\&= q_j^{uv\theta /e_j}(x) \left( \prod _{\begin{array}{c} i = 1 \\ i \ne j \end{array}}^d q_i^{uv\theta /e_i}(x)\right) \\&= q_j^{uv\theta /e_j}(x) \Big (1 + b(x) q_j^{uv/e_j}(x)\Big ) \end{aligned}$$
  for some polynomial $b(x) \in \mathbb {F}_q[x]$. Thus, here we also have
  $$\begin{aligned} C^{(j)}_{(m+1) \times (m+1)}\varDelta _{n,l} =\left( \underbrace{0, \dots , 0}_{uv\theta }, \phi ^{-1}(1),\underbrace{0, \dots , 0}_{uv-1}, a, \dots \right) ^\top , \end{aligned}$$
  with $$a \in \mathbb {F}_q$$.

Assume now that the $(uv\theta +1)$-st entry $y^{(1)}_{uv\theta +1}$ of $C^{(1)} \cdot (\phi ^{-1}(n_0), \phi ^{-1}(n_1), \dots )^\top$ fulfills

$$\begin{aligned}&|\phi (y^{(1)}_{uv\theta + 1} + \phi ^{-1}(1)) - \phi (y^{(1)}_{uv\theta + 1})| \\&\quad = \max \{|\phi (\alpha + \phi ^{-1}(1)) - \phi (\alpha )|: \text { for } \alpha \in \mathbb {F}_q\} =: w\ge 1, \end{aligned}$$

which is the case for at least $q^{m-1}$ many values of $n \in \{0, 1, \dots , q^m-1\}$. Then, for such *n* it holds that

8 $$\begin{aligned} \Vert \varvec{x}_n - \varvec{x}_l\Vert _\infty \in \Big [\frac{w}{q^{uv\theta +1}} - \frac{1}{q^{uv\theta + uv}}, \frac{w}{q^{uv\theta +1}} + \frac{1}{q^{uv\theta + uv}} \Big ). \end{aligned}$$

Finally, let $\varepsilon > 0$ and set

$$\begin{aligned} a = 2^{1/d}\frac{w}{q} - \varepsilon , \qquad b = 2^{1/d}\frac{w}{q} + \varepsilon , \qquad c = \frac{1}{q}. \end{aligned}$$

Thus, for $m = uv\theta d$, where *u* is a power of the characteristic of $\mathbb {F}_q$, we have

$$\begin{aligned} \#\Big \{0 \le n \ne l \le 2q^m -1 : \Vert \varvec{x}_n - \varvec{x}_l\Vert _{\infty } \in \Big (\frac{a}{(2q^m)^{1/d}},\frac{b}{(2q^m)^{1/d}}\Big ]\Big \} \ge c 2q^m \end{aligned}$$

provided that *u* is chosen large enough. However, since $\varepsilon$ can be chosen such that

$$\begin{aligned} (2b)^d - (2a)^d = 2^d\left( \left( 2^{1/d}\frac{w}{q} + \varepsilon \right) ^d - \left( 2^{1/d}\frac{w}{q} - \varepsilon \right) ^d \right) < \frac{1}{q} = c, \end{aligned}$$

the assumptions of Proposition 1 are fulfilled and the considered sequences therefore do not have Poissonian pair correlations. $\square$

### Application to Halton sequences

In order to be able to apply Proposition 1 to Halton sequences, we need a preliminary result, formulated as Lemma 3 below.

However, this lemma makes use of *Minkowski’s Theorem* (see [17]) that states that if $C \subseteq \mathbb {R}^d$ is a convex set that is symmetric about the origin (i.e., $x \in C$ if and only if $-x \in C$) and with $\mathrm{vol}(C) > 2^d m$, then there are at least *m* different points $\varvec{z}_1, \dots , \varvec{z}_m$ such that $\pm \varvec{z}_1, \dots , \pm \varvec{z}_m \in C \cap \mathbb {Z}^d {\setminus } \{\varvec{0}\}$.

#### Lemma 3

Let $$d \in \mathbb {N}$$ and $$\alpha _1, \dots , \alpha _d$$ be irrational. Then the sequence $$(\{n \varvec{\alpha }\})_{n\ge 0}$$ in $$[0,1)^d$$ with $$\{n \varvec{\alpha }\} = (\{n\alpha _1\}, \dots , \{ n\alpha _d\})$$ has an accumulation point in

$$\begin{aligned} D:= \{(\delta _1, \dots , \delta _d): \delta _j \in \{0,1\}, j \in \{1, \dots , d\}\}. \end{aligned}$$

#### Proof

For $N \in \mathbb {N}$ and arbitrary $\varepsilon _j > 0$, $j \in \{1, \dots , d\}$, define $C_N \in \mathbb {R}^{d+1}$,

$$\begin{aligned} C_N:=\{(x_0,x_1, \dots , x_d) \in \mathbb {R}^{d+1}: |\alpha _j x_0 - x_j| \le \varepsilon _j, j \in \{1, \dots , d\}, |x_0| \le N\}. \end{aligned}$$

The set *C* is convex and symmetric about the origin with

$$\begin{aligned} \text {vol}(C) = 2^{d+1} N \prod _{j = 1}^d\varepsilon _j. \end{aligned}$$

Therefore, if $N > m / (\prod _{j = 1}^d\varepsilon _j)$, we have $\text {vol}(C) > 2^{d+1} m$ and, by Minkowski’s Theorem, there exist *m* different elements $\varvec{z}_i = (z_i^{(0)}, z_i^{(1)}, \dots , z_i^{(d)})$, $i \in \{1, \dots , m\}$ with $\varvec{z}_i \in C \cap \mathbb {Z}^{d+1} {\setminus }\{\varvec{0}\}$ and $z_i^{(0)} \ge 0$. Moreover, for those elements it holds that $|\alpha _j z_i^{(0)} - z_i^{(j)}|\le \varepsilon _j$, thus $\{\alpha _j z_i^{(0)}\} \in (0,\varepsilon _j] \cup [1-\varepsilon _j,1)$ for all $j \in \{1, \dots , d\}$. Note that, if $\varepsilon _j$ are chosen small enough the integers $z_i^{(0)}$ will be distinct. $\square$

#### Proof of Theorem 2

For $d=1$ we have to consider the van der Corput sequence for which it is well-known that it does not have Poissonian pair correlations. Hence we assume $d\ge 2$ in the following. Let $b_1, \dots , b_d$ be pairwise relatively prime integers and let $(\varvec{x}_n)_{n \ge 0}$ denote the Halton sequence in bases $b_1, \dots , b_d$. Without loss of generality we assume $b_1 < b_j$ for all $j \in \{2, \dots , d\}$.

Let $u \in \mathbb {N}\setminus \{1\}$ and define

$$\begin{aligned} P_1&:= \prod _{j = 2}^{d}b_j^2, \qquad P_i := b_1^u\left( \prod _{\begin{array}{c} j = 2 \\ j \ne i \end{array}}^{d}b_j^2 \right) , \\ \tau _1&:= \min \{1 \le l \le P_1: b_1^{ul} \equiv 1 \pmod {P_1}\}, \\ \tau _i&:= \min \{1 \le l \le P_i: b_i^{2l} \equiv 1 \pmod {P_i}\} \end{aligned}$$

for all $i \in \{2, \dots , d\}$. Such $\tau _1, \tau _i$ exist as $\gcd (P_1,b_1)=\gcd (P_i,b_i)=1$ and $d\ge 2$.

Similar as in the proof of Theorem 1 we define for $\varvec{k} = (k_1, \dots , k_d)\in \mathbb {N}_0^d$ numbers $N_{\varvec{k}}\in \mathbb {N}$ and corresponding subintervals

$$\begin{aligned} I := I(u\tau _1k_1, 2\tau _2k_2, \dots , 2\tau _dk_d) =\left[ \frac{a_1}{b_1^{u\tau _1k_1}},\frac{a_1+1}{b_1^{u\tau _1k_1}}\right) \times \prod _{j=2}^d\left[ \frac{a_j}{b_j^{2\tau _jk_j}},\frac{a_j+1}{b_j^{2\tau _jk_j}}\right) , \end{aligned}$$

where $0 \le a_1 < b_1^{u\tau _1k_1}$ and $0\le a_j<b_j^{2\tau _jk_j}$ for $j \in \{2, \dots , d\}$, and study the distances between the points $\varvec{x}_n$ that lie in the same subinterval *I*.

Now let

$$\begin{aligned} M&= M(\varvec{k}) := b_1^{u\tau _1k_1} \left( \prod _{j = 2}^{d} b_j^{2\tau _j k_j}\right) , \\ L&= L(\varvec{k}) := b_1^{u\tau _1k_1+1} \left( \prod _{j = 2}^{d} b_j^{2\tau _j k_j+1}\right) . \end{aligned}$$

By a special regularity of the sequence, which is an easy consequence of the Chinese Remainder Theorem, we have that exactly $\prod _{j = 1}^{d}b_j$ points of the first *L* points and exactly one point of the subsequent *M* points of the sequence lie in *I*. Moreover, $\varvec{x}_{n+M}\in I$ if and only if $\varvec{x}_{n}\in I$.

We set $N_{\varvec{k}}:=L+M$ and study $\Vert \varvec{x}_n - \varvec{x}_{n + M}\Vert _\infty$ for $0\le n<L$.

By $(n)_{b_j}$ we denote the digit representation of *n* in base $b_j$, i.e. for $n = n_0 + n_1 b_j + n_2 b_j^2 + \dots$ we have $(n)_{b_j} = (n_0, n_1, n_2, \dots )$. Note that obviously $b_1^{u\tau _1k_1} |M$ and $b_j^{2\tau _jk_j}|M$. By the choice of $\tau _1$ and $\tau _j$ we have

$$\begin{aligned} b_1^{u\tau _1} \equiv 1 \pmod {b_j^2} \end{aligned}$$

and also for $i \ne j$,

$$\begin{aligned} b_i^{2\tau _i}\equiv 1 \pmod {b_j^{2}} \qquad \text { and } \qquad b_i^{2\tau _i}\equiv 1 \pmod {b_1^{u}}. \end{aligned}$$

Therefore,

$$\begin{aligned} \prod _{i = 2}^d b_i^{2\tau _ik_i} \equiv 1 \pmod {b_1^u} \qquad \text { and } \qquad b_1^{u\tau _1k_1}\prod _{i=2,i\ne j}^db_i^{2\tau _ik_i}\equiv 1 \pmod {b_j^{2}}. \end{aligned}$$

Hence,

9 $$\begin{aligned} \begin{aligned} (M)_{b_1}&= \left( \underbrace{0,\dots , 0}_{u\tau _1k_1}, 1, \underbrace{0, \dots , 0}_{u -1}, m_{u\tau _1 k_1 + u}, \dots \right) , \\ (M)_{b_j}&=\left( \underbrace{0,\dots , 0}_{2\tau _j k_j}, 1, 0, m_{2\tau _j k_j + 2}, \dots \right) ,\quad j\in \{2,\ldots ,d\}. \end{aligned} \end{aligned}$$

Now consider $\Vert \varvec{x}_n - \varvec{x}_{n + M}\Vert _\infty = \sup _{j \in \{1, \dots , d\}} \Vert x^{(j)}_n - x^{(j)}_{n + M}\Vert$. If for $(n)_{b_1}$ it holds that if $n_{u\tau _1 k_1} \ne b_1 -1$ then by (9) the first $u \tau _1 k_1 + u$ entries except of $(u \tau _1 k_1+1)$-st entry of $(n+M)_{b_1}$ and $(n)_{b_1}$ coincide. As $\sum _{i=m+1}^\infty \frac{b_1-1}{b_1^{i}}=\frac{1}{b_1^m}$ we have in the case where $n_{u \tau _1 k_1} \ne b_1-1$,

$$\begin{aligned} \Vert x^{(1)}_n - x^{(1)}_{n + M}\Vert \in \Big (\frac{1}{b_1^{u \tau _1k_1 +1}} - \frac{1}{b_1^{u \tau _1 k_1 + u}}, \frac{1}{b_1^{u \tau _1k_1 +1}} + \frac{1}{b_1^{u \tau _1 k_1 + u}}\Big ). \end{aligned}$$

Similarly, for the other coordinates $j \in \{2, \dots , d\}$ we obtain in the case where in $(n)_{b_j}$ we have $n_{2 \tau _j k_j} \ne b_j-1$,

$$\begin{aligned} \Vert x^{(j)}_n - x^{(j)}_{n + M}\Vert \in \Big (\frac{1}{b_j^{2 \tau _jk_j +1}} - \frac{1}{b_j^{2 \tau _j k_j + 2}}, \frac{1}{b_j^{2 \tau _jk_j +1}} + \frac{1}{b_j^{2 \tau _j k_j + 2}}\Big ). \end{aligned}$$

Next, we want to find constants $\xi _j\ge 1$, $j \in \{2, \dots , d\}$, such that

10 $$\begin{aligned} \xi _j \le \frac{b_j^{2\tau _jk_j +1}}{b_1^{u\tau _1k_1 +1}} \le \xi _j f(u) \end{aligned}$$

with

$$\begin{aligned} f(u) := \left( \frac{1+b_1^{1-u}}{1-b_1^{1-u}}\right) ^{\frac{d}{d-1}} \end{aligned}$$

is simultaneously fulfilled for infinitely many $\varvec{k} = (k_1, k_2, \dots , k_d) \in \mathbb {N}_0^d$ and thus also for infinitely many $N_{\varvec{k}} = M + L$. Therefore, we define $\beta _1 := b_1^{u \tau _1}$ and $\beta _j := b_j^{2\tau _j}$ for $j \in \{2, \dots , d\}$. The inequalities in (10) are then equivalent to

11 $$\begin{aligned} \log _{\beta _j}\Big (\xi _j\frac{b_1}{b_j}\Big ) + k_1 \log _{\beta _j}(\beta _1) \le k_j \le \log _{\beta _j}\Big (\xi _j f(u)\frac{b_1}{b_j}\Big ) + k_1 \log _{\beta _j}(\beta _1). \end{aligned}$$

Moreover, we consider the sequence $(\{n \varvec{\alpha }\})_{n\ge 0}\in [0,1)^{d-1}$ with $\{n \varvec{\alpha } \} = (\{n \alpha _2\}, \dots , \{n \alpha _d\})$ and $\alpha _j = \log _{\beta _j}(\beta _1) \in \mathbb {R}{\setminus }\mathbb {Q}$. Let now $(\delta _2, \dots , \delta _d) \in \{0,1\}^{d-1}$ denote an accumulation point of this sequence which exists by Lemma 3.

We want to distinguish two cases: If $\delta _j = 0$ we set

$$\begin{aligned} \xi _j := \frac{b_j}{b_1} \frac{1}{f(u)} \qquad \text { and } \qquad k_j := \lfloor k_1 \log _{\beta _j}(\beta _1)\rfloor . \end{aligned}$$

Note that $\xi _j > 1$ if *u* is large enough. The inequalities (11) are then equivalent to

$$\begin{aligned} \{k_1 \log _{\beta _j}(\beta _1)\} - \log _{\beta _j}(f(u)) \le 0 \le \{k_1 \log _{\beta _j}(\beta _1)\}, \end{aligned}$$

which is fulfilled if

12 $$\begin{aligned} \{k_1 \log _{\beta _j}(\beta _1)\} \in \big [0, \log _{\beta _j}(f(u))\big ]. \end{aligned}$$

If $\delta _j = 1$ we set

$$\begin{aligned} \xi _j := \frac{b_j}{b_1} \qquad \text { and } \qquad k_j := \lfloor k_1 \log _{\beta _j}(\beta _1)\rfloor +1. \end{aligned}$$

Again, $\xi _j > 1$ and (11) is equivalent to

$$\begin{aligned} \{k_1 \log _{\beta _j}(\beta _1)\} \le 1 \le \{k_1 \log _{\beta _j}(\beta _1)\} + \log _{\beta _j}(f(u)), \end{aligned}$$

which is fulfilled if

13 $$\begin{aligned} \{k_1 \log _{\beta _j}(\beta _1)\} \in \big [1-\log _{\beta _j}(f(u)),1\big ]. \end{aligned}$$

By the fact that $(\delta _2, \dots , \delta _d)$ is an accumulation point of $(\{n \varvec{\alpha }\})_{n\ge 0}$ with $\varvec{\alpha } = (\log _{\beta _2}(\beta _1), \dots , \log _{\beta _d}(\beta _1))$, conditions (12) and (13), respectively, are fulfilled simultaneously for each $j = 2, \dots , d$ for infinitely many $k_1$. Since $k_j \ge 0$ for all $j \in \{2, \dots , d\}$, we know that there are also infinitely many $N_{\varvec{k}}$ such that (10) is fulfilled.

Now we can use (10) to deduce that

$$\begin{aligned} \frac{1}{b_1^{u \tau _1 k_1 + 1}} - \frac{1}{b_1^{u \tau _1 k_1 + u}} > \frac{1}{b_j^{2 \tau _j k_j + 1}} + \frac{1}{b_j^{2 \tau _j k_j + 2}} \end{aligned}$$

for all $j \in \{2, \dots , d\}$ and *u* large enough. This can be seen since

$$\begin{aligned} \frac{b_j^{2 \tau _j k_j + 1}}{b_1^{u \tau _1 k_1 + 1}}\Big (1 - \frac{1}{b_1^{u -1}}\Big )&\ge \xi _j \Big (1 - \frac{1}{b_1^{u -1}}\Big ) \\&\ge \frac{b_j}{b_1} \frac{1}{f(u)}\Big (1 - \frac{1}{b_1^{u-1}}\Big ) \\&> \Big (1 + \frac{1}{b_j}\Big ), \end{aligned}$$

where in the last step we used that $(1-1/b_1^{u-1})/f(u) \rightarrow 1$ as $u \rightarrow \infty$, and $b_j-1 \ge b_1$. Therefore, if in $(n)_{b_1}$ we have that $n_{u \tau k_1} \ne b_1 -1$ and in $(n)_{b_j}$, $j \in \{2, \dots , d\}$ we have that $n_{2\tau _j k_j} \ne b_j-1$, then

14 $$\begin{aligned} \Vert \varvec{x}_n - \varvec{x}_{n+M}\Vert _\infty = \Vert x_n^{(1)} - x_{n+M}^{(1)}\Vert \in \Big (\frac{1-b_1^{1-u}}{b_1^{u \tau _1 k_1 +1}},\frac{1+b_1^{1-u}}{b_1^{u \tau _1 k_1 +1}}\Big ). \end{aligned}$$

As next step, we want to establish suitable bounds for $\Vert \varvec{x}_n - \varvec{x}_{n+M}\Vert _\infty$ in order to be able to apply Proposition 1, i.e. we want to show that there exist *a* and *b* such that

$$\begin{aligned} \frac{a}{(L+M)^{1/d}} \le \frac{1-b_1^{1-u}}{b_1^{u \tau _1 k_1 +1}} < \frac{1+b_1^{1-u}}{b_1^{u \tau _1 k_1 +1}} \le \frac{b}{(L+M)^{1/d}}, \end{aligned}$$

which is equivalent to

15 $$\begin{aligned} a^d \le \frac{(1-b_1^{1-u})^d (L+M)}{(b_1^{u \tau _1 k_1 +1})^d} < \frac{(1+b_1^{1-u})^d (L+M)}{(b_1^{u \tau _1 k_1 +1})^d} \le b^d. \end{aligned}$$

Note that

$$\begin{aligned} L+M = b_1^{u \tau _1 k_1 +1}\left( \prod _{j = 2}^{d} b_j^{2\tau _j k_j+1}\right) \underbrace{\left( 1 + \prod _{j = 1}^{d} b_j^{-1}\right) }_{:=\gamma ^d}=L\gamma ^d. \end{aligned}$$

Using the estimate (10) we find that (15) is fulfilled if we choose

$$\begin{aligned} a^d&:= (1 - b_1^{1-u})^d \left( \prod _{j = 2}^d\xi _j\right) \gamma ^d, \\ b^d&:= \frac{(1 + b_1^{1-u})^{2d}}{(1 - b_1^{1-u})^d} \left( \prod _{j = 2}^d\xi _j\right) \gamma ^d. \end{aligned}$$

Hence we have shown that

$$\begin{aligned} \Vert \varvec{x}_n - \varvec{x}_{n+M}\Vert _\infty \in \Big (\frac{a}{(L+M)^{1/d}}, \frac{b}{(L+M)^{1/d}}\Big ) \end{aligned}$$

for $n \in \{0, \dots , L -1\}$ whenever in $(n)_{b_1}$ we have $n_{u \tau k_1} \ne b_1 -1$ and in $(n)_{b_j}$, $j \in \{2, \dots , d\}$ we have $n_{2\tau _j k_j} \ne b_j-1$. Since this is the case for exactly $\big (\prod _{j = 1}^{d} \frac{b_j-1}{b_j}\big )L$ values of *n* and each pair has to be counted twice in the pair correlation function, we obtain

$$\begin{aligned} \#\Big \{0 \le n&\ne l \le L+M-1: \Vert \varvec{x}_n - \varvec{x}_l\Vert _\infty \in \Big (\frac{a}{(L+M)^{1/d}}, \frac{b}{(L+M)^{1/d}}\Big ]\Big \} \\&\ge \#\Big \{0 \le n \le L-1: \Vert \varvec{x}_n - \varvec{x}_{n+M}\Vert _\infty \in \Big (\frac{a}{(L+M)^{1/d}}, \frac{b}{(L+M)^{1/d}}\Big ]\Big \} \\&\ge 2 \Big (\prod _{j = 1}^{d}\frac{b_j-1}{b_j}\Big )\frac{L}{L+M} (L+M) \\&= 2 \Big (\prod _{j = 1}^{d}\frac{b_j-1}{b_j}\Big ) \frac{1}{\gamma ^d}(L +M)=:c\cdot (L+M). \end{aligned}$$

In order to apply Proposition 1 it therefore has to hold that

16 $$\begin{aligned} (2b)^d - (2a)^d < 2 \left( \prod _{j = 1}^{d}\frac{b_j-1}{b_j}\right) \frac{1}{\gamma ^d}=c. \end{aligned}$$

Using the definition of *a* and *b* and the fact that $\xi _j \le b_j /b_1$ we obtain

$$\begin{aligned} (2b)^d - (2a)^d&= 2^d \left( \prod _{j = 2}^d\xi _j\right) \gamma ^d \Big (\frac{(1+b_1^{1-u})^{2d}}{(1-b_1^{1-u})^d} - (1- b_1^{1-u})^d\Big ) \\&\le 2^d \left( \prod _{j = 2}^d\frac{b_j}{b_1}\right) \gamma ^d \underbrace{\Big (\frac{(1+b_1^{1-u})^{2d} - (1- b_1^{1-u})^{2d}}{(1-b_1^{1-u})^d}\Big )}_{\text {tends to 0 for } u \rightarrow \infty }. \end{aligned}$$

Thus, if *u* is chosen large enough, condition (16) is true and the Halton sequence in bases $b_1, \dots , b_d$ does not have Poissonian pair correlations. $\square$

## Discussion and further research

It is an interesting task for future research to figure out whether the Halton and the Niederreiter sequence do have Poissonian pair correlations in the sense of [22] or not. Of course we conjecture that both do not have this type of Poissonian pair correlations. Both proofs of our Theorems 1 and 2 use specific pairs of points $(\varvec{x}_n,\varvec{x}_l)$ such that $\Vert \varvec{x}_n-\varvec{x}_l\Vert _{\infty }$ can be estimated very well (cf. (8) and (14)). A possible generalization of our Theorems 1 and 2 to the notion of Poissonian pair correlation in [22], based on our method of proof will rely on sharp estimates of $\Vert \varvec{x}_n-\varvec{x}_l\Vert _{2}$ on a sufficiently large number of pairs $(\varvec{x}_n,\varvec{x}_l)$.

However, we suggest the following alternative general approach that is to answer the basic question whether Poissonian pair correlation based on the supremum norm implies Poissonian pair correlation based on the Euclidean norm and vice versa.

Theorem 1 of the present paper deals with very prominent classes of (*t*, *s*)-sequences, as a consequence of this result, of course a further research question is, whether other classes of (*t*, *s*)-sequences, as for example generalized Niederreiter sequences [23], Niederreiter–Xing sequences [26] or any digital or even non-digital (*t*, *s*)-sequences in general, have the property of Poissonian pair correlation or not.

Furthermore, we would like to note an interesting relation of our method of proof to a conjecture in algebraic and transcendental number theory. During the search for a proof of Theorem 2 we faced the problem to simultaneously satisfy the inequalities (11) with $\xi _j\ge 1$ for infinitely many $(k_1,k_2,\ldots ,k_d)\in \mathbb {N}_0^d$.

Note that if $$1,\log _{\beta _2}\beta _1,\ldots ,\log _{\beta _d}\beta _1$$ were linearly independent over $$\mathbb {Q}$$ then the sequence $$(\{n(\log _{\beta _2}\beta _1,\ldots ,\log _{\beta _d}\beta _1)\})_{n\ge 0}\in [0,1)^{d-1}$$ would be uniformly distributed in $$[0,1)^{d-1}$$. Such a statement would considerably shorten the proof of Theorem 2. Unfortunately, it is not known whether for example the three numbers $$1/\log 2,1/\log 3,1/\log 5$$ are linearly independent over $$\mathbb {Q}$$ or not. The algebraic independence of the logarithm of the prime numbers would be one consequence of the so-called Schanuel’s conjecture in algebraic and transcendental number theory. We refer the interested reader to [25] for more details on this conjecture and its related problems.

## References

[1] Aichinger, I., Aistleitner, C., Larcher, G.: On Quasi-Energy-Spectra, Pair Correlations of Sequences and Additive Combinatorics. In: Contemporary Computational Mathematics—A Celebration of the 80th Birthday of Ian Sloan, pp. 1–16. Springer International Publishing, Cham (2018)

[2] Aistleitner C, Lachmann T, Pausinger F (2018). Pair correlations and equidistribution. J. Number Theory.

[3] Dick J, Pillichshammer F (2010). Digital Nets and Sequences: Discrepancy Theory and Quasi-Monte Carlo Integration.

[4] Faure H (1982). Discrépance de suites associées à un système de numération (en dimension $$s$$). Acta Arith..

[5] Faure H, Kritzer P, Pillichshammer F (2015). From van der Corput to modern constructions of sequences for quasi-Monte Carlo rules. Indag. Math. New Ser..

[6] Faure H, Tezuka S, Fang KT, Niederreiter H, Hickernell FJ (2002). Another random scrambling of digital (t, s)-sequences. Monte Carlo and Quasi-Monte Carlo Methods 2000.

[7] Grepstad S, Larcher G (2017). On pair correlation and discrepancy. Archiv der Mathematik.

[8] Halton JH (1960). On the efficiency of certain quasi-random sequences of points in evaluating multi-dimensional integrals. Numerische Mathematik.

[9] Hinrichs A, Kaltenböck L, Larcher G, Stockinger W, Ullrich M (2019). On a multi-dimensional Poissonian pair correlation concept and uniform distribution. Monatshefte für Mathematik.

[10] Hofer R (2013). A construction of low-discrepancy sequences involving finite-row digital $$(t, s)$$-sequences. Monatshefte für Mathematik.

[11] Hofer R, Niederreiter H (2013). A construction of (t, s)-sequences with finite-row generating matrices using global function fields. Finite Fields Appl..

[12] Hofer R, Pirsic G, Dick J, Kuo FY, Peters GW, Sloan IH (2013). A finite-row scrambling of Niederreiter sequences. Monte Carlo and Quasi-Monte Carlo Methods 2012.

[13] Larcher, G., Stockinger, W.: On pair correlation of sequences. In: Discrepancy theory, pp. 133–145. Berlin: De Gruyter (2020)

[14] Larcher G, Stockinger W (2020). Some negative results related to Poissonian pair correlation problems. Discrete Math..

[15] Levin, M.B.: On the upper bound of the $$L_p$$ discrepancy of Halton’s sequence and the Central Limit Theorem for Hammersley’s net (2018). arXiv:1806.11498

[16] Marklof J (2020). Pair correlation and equidistribution on manifolds. Monatsh. Math..

[17] Minkowski H (1953). Geometrie der Zahlen.

[18] Niederreiter H (1988). Low-discrepancy and low-dispersion sequences. J. Number Theory.

[19] Niederreiter H (1992). Random Number Generation and Quasi-Monte Carlo Methods.

[20] Pillichshammer F (2004). On the discrepancy of $$(0,1)$$-sequences. J. Number Theory.

[21] Steinerberger S (2017). Localized quantitative criteria for equidistribution. Acta Arithmetica.

[22] Steinerberger S (2020). Poissonian pair correlation in higher dimensions. J. Number Theory.

[23] Tezuka S (1993). Polynomial Arithmetic Analogue of Halton Sequences. ACM Trans. Model. Comput. Simul..

[24] Tezuka S (2013). On the discrepancy of generalized Niederreiter sequences. J. Complex..

[25] Waldschmidt, M.: Diophantine Approximation on Linear Algebraic Groups: Transcendence Properties of the Exponential Function in Several Variables, Grundlehren der mathematischen Wissenschaften, vol. 326. Springer, Berlin, Heidelberg (2000) 10.1007/978-3-662-11569-5

[26] Xing C, Niederreiter H (1995). A construction of low-discrepancy sequences using global function fields. Acta Arithmetica.

